# Editorial: Neuroimaging Biomarkers in Mood and Anxiety Disorders

**DOI:** 10.3389/fpsyt.2021.773034

**Published:** 2021-10-29

**Authors:** Chien-Han Lai, Yong-Ku Kim, Joaquim Radua

**Affiliations:** ^1^Institute of Biophotonics, National Yang-Ming University, Taipei, Taiwan; ^2^PhD Psychiatry & Neuroscience Clinic, Taoyuan City, Taiwan; ^3^Korea University, Seoul, South Korea; ^4^Imaging of Mood- and Anxiety-Related Disorders (IMARD) Group, Institut d'Investigacions Biomèdiques August Pi i Sunyer (IDIBAPS), Centro de Investigación Biomédica en Red de Salud Mental (CIBERSAM), Barcelona, Spain; ^5^Institute of Psychiatry, Psychology, and Neuroscience, King's College London, London, United Kingdom; ^6^Department of Clinical Neuroscience, Karolinska Institute, Stockholm, Sweden

**Keywords:** depression, anxiety, MRI, MEG, TMS

For the coming new era of the modern world, accumulating stressors will represent a significant hurdle, likely increasing the prevalence of mood and anxiety disorders. Furthermore, the consequences of the COVID-19 pandemic may further increase these disorders. Thus, we need to investigate potential biomarkers for these disorders, as they could assist us in alleviating the clinical symptoms and preventing the subsequent sequela. Since we know that the brain controls and manipulates psychological status and related behaviors, we should scrutinize the brain's biomarkers of mood and anxiety disorders.

There are many methods to study our brain. Still, from the translational and non-invasive perspectives, the neuroimaging method is an ideal option. In this Research Topic, we included seven articles about the neuroimaging investigation of mood and anxiety disorders (Ballard et al.; Zhou et al.; Zhang Y. et al.; Zhang T. et al.; Zhang J. et al.; Peräkylä et al.; Oh et al.). Their neuroimaging methods included magnetoencephalography (MEG; Ballard et al.), functional and structural magnetic resonance imaging (MRI; Zhou et al.; Zhang Y. et al.; Zhang J. et al.; Oh et al.), repetitive transcranial magnetic stimulation (r-TMS; Zhang T. et al.), and electroconvulsive therapy (ECT; Peräkylä et al.). The mood and anxiety conditions discussed in the seven articles of this Research Topic consisted of suicide crisis (Ballard et al.), bipolar II depression (Zhou et al.), major depressive disorder (MDD; Zhang Y. et al.; Zhang T. et al.; Zhang J. et al.; Peräkylä et al.), and obsessive-compulsive disorder (OCD; Oh et al.). Four articles investigated depression-related brain alterations (Ballard et al.; Zhou et al.; Zhang Y. et al.; Peräkylä et al.).

One work in this Research Topic found that the amygdala and insula connectivity alterations may play a role in implicit suicidal associations (Ballard et al.). The connectivity estimates between the early visual cortex, anterior insula, and amygdala may identify the suicide crisis patients with acceptable sensitivity and specificity. Another work found the slow five fractional amplitude of low-frequency fluctuations associated with bipolar II depression symptoms in several clusters (Zhou et al.). For MDD, one article found reductions of gray matter volume in the fronto-limbic-striatal regions, including the prefrontal lobe, limbic system, striatum, cerebellum, temporal lobe, and bilateral lingual gyri (Zhang Y. et al.). A strength of that study is that it only included first-episode medicine-naive MDD patients.

Another article used rTMS with different frequency parameters in the dorsolateral prefrontal cortex within a randomized double-blinded controlled trial (Zhang T. et al.). The study found no statistical differences in the efficacy of rTMS between unilateral left and bilateral DLPFC and between 5 and 10 Hz for treating MDD. The aim of another article was to find possible therapeutic effects for acupuncture in MDD (Zhang J. et al.). It found that the potential effects might be limited to the functional MRI perspectives, such as increased functional connectivity of the anterior cingulate cortex. Conversely, it did not identify significant neurological changes in any DTI or VBM studies after acupuncture treatment in MDD. Yet another work about MDD in this topic used novel indices derived from threat modulation (Peräkylä et al.). The authors found that executive function and working memory indicators may be potential objective biomarkers of depression severity before ECT and cognitive outcome after ECT.

The only article not related to depression in this Research Topic focused on OCD. The researchers used a mental rotation task to assess whether activation in the right dorsolateral prefrontal cortex in patients with OCD was positively associated with clinical severity (Oh et al.). OCD patients could activate the frontoparietal regions like healthy controls during the task, but the results indicated the possible specific pattern of alterations of OCD patients.

With such rich content and broad coverage in the discussion of neuroimaging findings in MDD and OCD, we hope you have a great journey and harvest when you enjoy the exciting and meaningful results in the articles of this Research Topic.

## Author Contributions

C-HL drafted the manuscript. JR and Y-KK advised and revised the manuscript. All authors contributed to the article and approved the submitted version.

## Conflict of Interest

The authors declare that the research was conducted in the absence of any commercial or financial relationships that could be construed as a potential conflict of interest.

## Publisher's Note

All claims expressed in this article are solely those of the authors and do not necessarily represent those of their affiliated organizations, or those of the publisher, the editors and the reviewers. Any product that may be evaluated in this article, or claim that may be made by its manufacturer, is not guaranteed or endorsed by the publisher.

